# Two-step cleavage of hairpin RNA with 5' overhangs by human DICER

**DOI:** 10.1186/1471-2199-12-6

**Published:** 2011-02-09

**Authors:** Yoshinari Ando, Yoshiko Maida, Ayako Morinaga, Alexander M Burroughs, Ryuichiro Kimura, Joe Chiba, Harukazu Suzuki, Kenkichi Masutomi, Yoshihide Hayashizaki

**Affiliations:** 1 RIKEN Omics Science Center, 1-7-22 Suehiro-cho, Tsurumi-ku, Yokohama 230-0045, Japan; 2 Cancer Stem Cell Project, National Cancer Center Research Institute, 5-1-1 Tsukiji, Chuo-ku, Tokyo 104-0045, Japan; 3 Department of Biological Science and Technology, Tokyo University of Science, 2641 Yamazaki, Noda, Chiba 278-8510, Japan; 4 PREST, Japan Science and Technology Agency, 4-1-8 Honcho Kawaguchi, Saitama 332-0012, Japan

## Abstract

**Background:**

DICER is an RNase III family endoribonuclease that processes precursor microRNAs (pre-miRNAs) and long double-stranded RNAs, generating microRNA (miRNA) duplexes and short interfering RNA duplexes with 20~23 nucleotides (nts) in length. The typical form of pre-miRNA processed by the Drosha protein is a hairpin RNA with 2-nt 3' overhangs. On the other hand, production of mature miRNA from an endogenous hairpin RNA with 5' overhangs has also been reported, although the mechanism for this process is unknown.

**Results:**

In this study, we show that human recombinant DICER protein (rDICER) processes a hairpin RNA with 5' overhangs *in vitro *and generates an intermediate duplex with a 29 nt-5' strand and a 23 nt-3' strand, which was eventually cleaved into a canonical miRNA duplex via a two-step cleavage. The previously identified endogenous pre-miRNA with 5' overhangs, pre-mmu-mir-1982 RNA, is also determined to be a substrate of rDICER through the same two-step cleavage.

**Conclusions:**

The two-step cleavage of a hairpin RNA with 5' overhangs shows that DICER releases double-stranded RNAs after the first cleavage and binds them again in the inverse direction for a second cleavage. These findings have implications for how DICER may be able to interact with or process differing precursor structures.

## Background

DICER plays a key role in RNA interference pathways through the biogenesis of microRNA (miRNA) and small interfering RNA (siRNA) [1-3]. Most miRNA genes are transcribed as long primary transcripts (pri-miRNAs) where stem-loop structures with mature miRNA sequences embedded in the arm of a stem are cleaved by the Drosha nuclear microprocessor complex releasing a precursor miRNA (pre-miRNA) hairpin [4,5]. The cleavage site is determined mainly by the distance (~11 bp) from the stem-single stranded RNA junction of pri-miRNA and most pre-miRNAs have 2 nt-3' overhangs [6]. Pre-miRNAs, exported into the cytoplasm by Exportin-5 and Ran-GTP [7], are processed by the RISC loading complex (RLC) into 20~23 nt duplexes where the RNase III enzyme DICER plays a central role together with the double stranded (ds) RNA-binding proteins TRBP and PACT and the miRNA-associated RNA-induced silencing complex (miRISC) core component Argonaute-2 (AGO2) [8-10]. miRNA duplexes processed by RLC are finally loaded to miRISC as a double stranded-structure [11] and separated into the functional guide strand, which is complementary to the target, and the passenger strand, which is subsequently degraded [12,13]. Strand selection of the functional guide strand by AGO2 depends on the thermodynamic stabilities of the base pairs at the 5' ends of the two strands [12,14,15]. Duplexes of siRNA or miRNA produced by DICER can be loaded in either direction to Argonaute [16-18]. Indeed, the mature miRNA either in the 5' or 3' strands can be harboured from pre-miRNA [19-21]. On the other hand, endogenous human AGO2 can bind directly to pre-miRNAs in DICER-knockout cells [22]. Recently, it was reported that human DICER is not essential for loading dsRNAs to AGO2 but functions in pre-selection of effective siRNAs for handoff to AGO2 [23].

Human DICER is a ~220 kDa protein consisting of several domains; an N-terminal DExH-box RNA helicase-like domain, a DUF283 domain, a PAZ domain, two RNase III domains (RIIIa and RIIIb), and a dsRNA binding motif domain (DARM) [24]. The two RNase III domains of DICER form a single dsRNA processing center via intramolecular dimerization which together cleave the opposite strands of the dsRNA, generating dinucleotide-long 3' overhangs on both ends [25]. The crystal structure of Dicer from *Giardia intestinalis *showed that the hydrophobic pocket of the PAZ domain was responsible for the binding of the 3' dinucleotide overhangs of the substrate and the connector helix between the PAZ domain and RNase III domain functioned as a molecular ruler measuring the distance from the 3' end of pre-miRNA to the cleavage site [26,27]. However, 3'-dinucleotide dsRNA overhangs are not essential for binding with DICER [28]. When the 3' overhang is removed, DICER can still cleave dsRNA through interaction with the remaining 5' overhang [28]. This is consistent with MacRae *et al. *who found that the recombinant Dicer protein of *Giardia intestinalis *could cleave the dsRNA with 5' overhangs [27]. However, they used perfectly matched dsRNAs with no gap, which might resemble an endogenous siRNA precursor. An additional study by Flores-Jasso *et al. *showed that human recombinant DICER protein could nick either strand of a mononucleotide-5' overhanged pre-miRNA with some strand preferences [29]. Despite this, the detailed step mechanism for pre-miRNA cleavage, especially for the pre-miRNA with 5' overhangs, is not yet elucidated.

An alternative nuclear pathway of pre-miRNA biogenesis was described where a short intron with a hairpin can be spliced and debranched into pre-miRNA hairpin mimics (mirtrons) [30-32]. This processing pathway uses intron splicing machinery instead of the Drosha endonuclease; miRNA precursors generated from intronic sequences (debranched mirtrons) are believed to be incorporated into the canonical miRNA pathway as a substrate of DICER. Interestingly, mouse pre-mir-1982 is a mirtron with an 11 nt tail at the 5' end [33], although most mammalian mirtron are hairpin structures with single nucleotide overhangs at both ends [32-34]. Mature mouse miR-1982* miRNA emerges without 11 nt-5' overhangs from deep sequencing data of murine cells [33,35] while the elimination mechanism of this 11 nt-5' tail is still unknown.

In this paper, we investigated the detailed processing pattern of hairpin RNAs containing 5' overhangs by human recombinant DICER. We show here that human recombinant DICER is able to process hairpin RNA with 5' overhangs and two-step cleavage by DICER forms the mature miRNA duplex from the hairpin RNAs. Additionally, pre-mmu-mir-1982 RNA, which is a natural hairpin RNA with 5' overhangs, is also processed by a two-step cleavage mediated by human recombinant DICER protein *in vitro*.

## Results and Discussion

### Processing of the pre-miRNA by recombinant DICER protein

**Figure 1:**
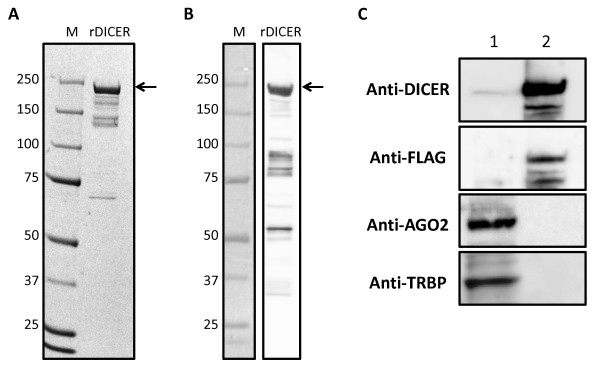
**Characterization of recombinant DICER protein**. Purified rDICER (3 μg) was loaded onto a NuPAGE 4-12% Bis-Tris gel. Proteins were analyzed by Coomassie Brilliant Blue (CBB) staining (**A**) and Western blotting with anti-DICER antibody (**B**). (**C**) Western blotting with anti-DICER, anti-FLAG, anti-AGO2 and anti-TRBP antibodies. 30 μg of 293T cell lysate (lane 1) and 3 μg of rDICER (lane 2) were loaded.

**Figure 2:**
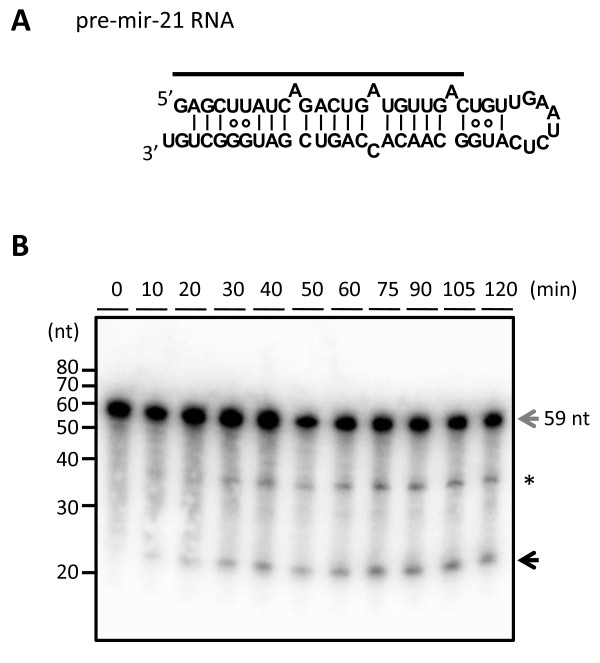
**Processing of the precursor miRNA by recombinant DICER protein**. (**A**) Precursor miRNA (pre-mir-21 RNA) and probe used in this study. The 5' end nucleotide of pre-mir-21 RNA was modified (U to G) from human pre-hsa-mir-21 RNA sequence registered in miRBase 14.0 [46-48] in order to facilitate the *in vitro *transcription reaction. The secondary structure was predicted using the CentroidFold program [49]. The solid line shows the position of probe-1. (**B**) Detection of the rDICER-processed products by Northern blotting. Pre-mir-21 RNAs were incubated with rDICER *in vitro *for the indicated time points (0, 10, 20, 30, 40, 50, 60, 75, 90, 105 and 120 min). The RNAs processed by rDICER were detected using probe-1 by Northern blotting. The gray arrow shows the band of pre-mir-21 RNA and the black arrow shows the band of miRNA processed from the 5' strand of pre-mir-21 RNA. The asterisk shows the nicked product (~37 nt) similar to a previous report [29].

We prepared purified recombinant DICER1 (rDICER) protein containing a FLAG-tag at the N-terminus (see Figures 1A and 1B). This rDICER does not contain known DICER-binding partners, AGO2 and TRBP (see Figure 1C). In order to confirm activity, we attempted to cleave pre-miRNA hairpin RNA using the rDICER. Forty-five pmol of pre-mir-21 RNA (see Figure 2A) was incubated with 2 pmol of the purified rDICER at the indicated times followed by purification. The reacted RNA substrates were subjected to Northern blotting using probe-1, corresponding to the antisense sequence of bases 2-22 of pre-mir-21 (see Figure 2A). A single band, 23 nucleotides in length, appeared after 20 min incubation and gradually increased. Thus, the purified rDICER possessed reasonable pre-miRNA processing activity to produce ~23 nt mature miRNA *in vitro *(see Figure 2B).

### Processing of the hairpin RNA with 5' overhangs, RNA-I, by recombinant DICER protein

**Figure 3:**
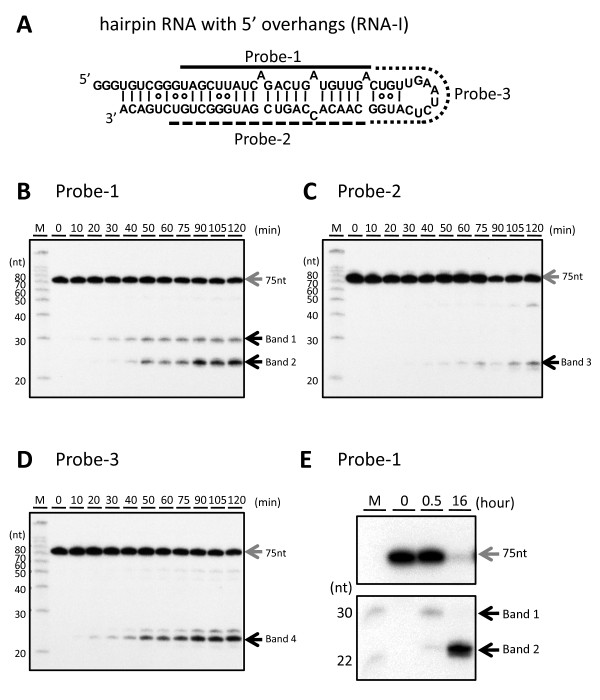
**Processing of the hairpin RNA with 5' overhangs by recombinant DICER protein**. (**A**) Hairpin RNA with 5' overhangs (RNA-I) and probes used in this study. RNA-I was a hairpin RNA with 5' overhangs based on the "pre-mir-21 RNA" sequence. The secondary structure was predicted using the CentroidFold program [49]. The solid line shows the position of probe-1, the dashed line shows the position of probe-2 and the dotted line shows the position of probe-3. (**B-D**) Time-course analysis of the processing of RNA-I by the rDICER protein. RNA-I RNAs were incubated with rDICER *in vitro *for the indicated time points (0, 10, 20, 30, 40, 50, 60, 75, 90, 105 and 120 min). The RNAs processed by rDICER were detected using probe-1, probe-2 and probe-3 (**B-D**, respectively) by Northern blotting. The gray arrow shows the band of unprocessed RNA-I and the black arrow shows the bands of small RNA processed from the 5' strand, 3' strand and loop region of RNA-I respectively. M: decade marker. (**E**) The processing of RNA-I by the rDICER protein at a longer incubation time. RNA-I RNAs were incubated with rDICER for 30 min and 16 hours. The RNAs processed from the 5' strand of RNA-I were detected using probe-1 by Northern blotting.

Using this rDICER, we performed a cleavage assay on a designed pre-miRNA mimic of hairpin RNA with trinucleotide-5' overhangs (RNA-I, see Figure 3A) to analyze whether DICER could process a hairpin RNA with 5' overhangs. The cleavage products were detected by Northern blotting using three different probes, probe-1, probe-2 and probe-3, corresponding to antisense sequences of bases 11-32, bases 49-69 and bases 33-48 of RNA-I, respectively (see Figure 3A). Using probe-1, band 1 (~30nt) appeared after 20 min incubation and gradually increased through a 50 min-incubation. This processing pattern was similar to that of the around 23-nt product generated from native pre-miRNA by rDICER (see Figures 2B and 3B). After 40 min incubation, band 2 (~23nt) was detected and the abundance of band 2 increased in a time-dependent manner (see Figure 3B). Additionally, using probe-2, band 3 (~23nt) was detected from the 30-min incubation sample (see Figure 3C). Using probe-3, band 4 (~22nt) was detected from the 20-min incubation sample (see Figure 3D). The processing activity on the hairpin RNA with 5' overhangs is comparable to that for natural pre-miRNA. This means that hairpin RNA with 5' overhangs could also be a substrate for rDICER processing.

To analyze band 1 at a longer incubation time, the RNA-I was incubated with the purified rDICER and the cleavage reactions were performed for 30 min and 16 hours. Surprisingly, band 1 was detected at 30 min incubation but disappeared after 16-hours incubation. On the other hand, band 2 continued to accumulate (see Figure 3E). These results showed that band 1, which seems to be a product of first processing by rDICER, disappeared following an extended incubation time.

Next, to verify the size of the cleavage products of RNA-I, we cloned the 23-nt products after 16-hour rDICER incubation and sequenced them (see Table 1). Several clones were obtained from 5'-strand, 3' strand and loop region of RNA-I corresponding to bands 2-4 in Figure 3, respectively. The miRNA length heterogeneity generated by rDICER is consistent with the finding in the previous report [36]. Clones from the 5' strand lacking 6 or 7 nt the initial of the 5' end of RNA-I and clones from 3' strand lack the terminal 1 or 2 nt of the 3' end of RNA-I.

**Table 1:** Cloning of products from RNA-I processed by recombinant DICER protein

	Count
......CGGGUAGCUUAUCAGACUGAUGU..............................................	23
.......GGGUAGCUUAUCAGACUGAUGU..............................................	19
......CGGGUAGCUUAUCAGACUGAUG...............................................	9
.....UCGGGUAGCUUAUCAGACUGAUGU..............................................	1
.....UCGGGUAGCUUAUCAGACUGAUG...............................................	1
.......GGGUAGCUUAUCAGACUGAUGUU.............................................	1
......CGGGUAGCUUAUCAGACUGA.................................................	1
.............................UGACUGUUGAAUCUCAUGGCAA........................	4
.............................UGACUGUUGAAUCUCAUGGCAACA......................	2
............................UUGACUGUUGAAUCUCAUGGCAAC.......................	1
....................................................ACCAGUCGAUGGGCUGUCUGAC.	2
.....................................................CCAGUCGAUGGGCUGUCUGA..	2
....................................................ACCAGUCGAUGGGCUGUCUGA..	1

GGGUGUCGGGUAGCUUAUCAGACUGAUGUUGACUGUUGAAUCUCAUGGCAACACCAGUCGAUGGGCUGUCUGACA	
...((((((((((((((((.(((((.(((((.((((........))))))))).)))))))))))))))))))))	(-34.60)

**Figure 4:**
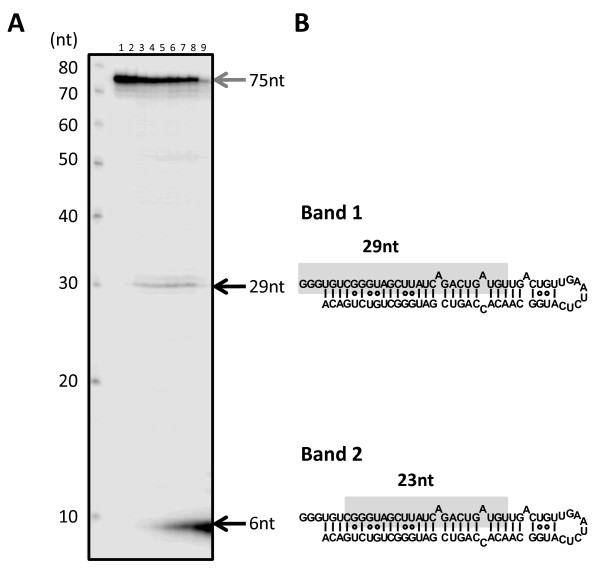
**Two-step processing of the 5'-end labelled RNA-I by recombinant DICER protein**. (**A**) *In vitro *processing of the 5'-end labelled RNA-I by rDICER protein. 5'-end labelled RNA-I RNAs were incubated without rDICER for 120 min (lane 1) and with rDICER for the following time points (0, 10, 20, 30, 40, 50, 60 and 120 min; lane 2-9 respectively). The processing reaction was faster than the results of Figure 3B because the amount of RNA substrate in this reaction mixture was less. The RNA products less than 10 nt look stacked at the end of the gel because of the difficulty in separating efficiently, even at 7.5 M urea denaturing 20% polyacrylamide sequence gel. This experiment was repeated and replicated consistently. M: decade marker. (**B**) Sequences of band 1 and 2 in Figure 3B identified from Figure 4A and Table 1. Sequences highlighted in gray are 29-nt (band 1) and 23-nt RNA (band 2) from the 5' strand, respectively.

**Figure 5:**
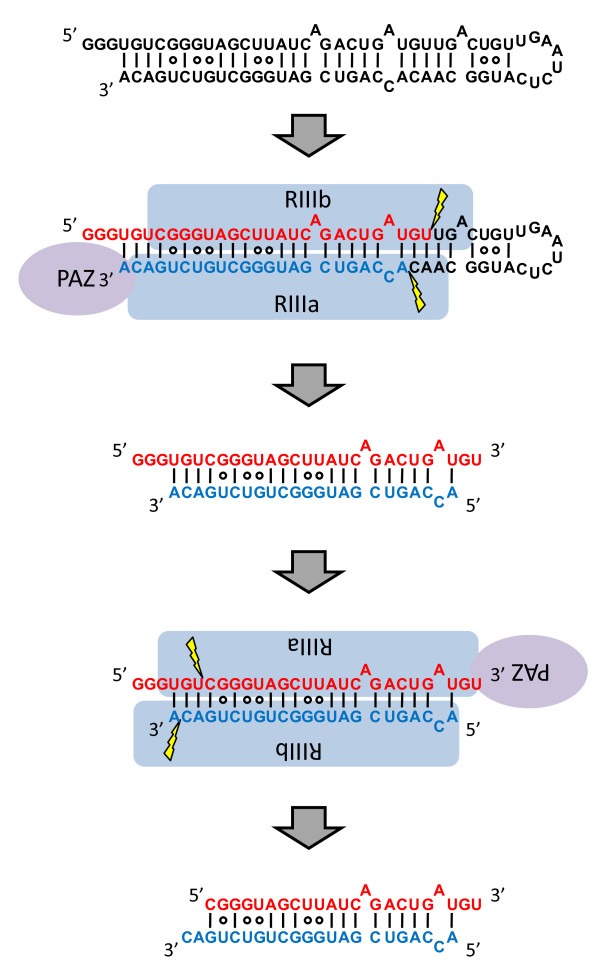
**Model of the two-step processing of hairpin RNA with 5' overhangs (RNA-I) by DICER protein**. rDICER processes hairpin RNA with 5' overhangs (RNA-I) to dsRNA with 29 nt-5' strand and 23 nt-3' strand after the first cleavage reaction and releases once from the binding site. Then, the dsRNA is bound in the inverse direction with the same or different rDICER molecule and is measured after the anchoring 3' end of the 29-nt strand to generate dsRNA with 23 nt cleaved from the 29-nt strand and 22 nt cleaved from the 23-nt strand. "PAZ" domain of rDICER colored purple; "RIIIa" and "RIIIb" domain of rDICER colored blue. Lightning marks indicate the cleavage sites in the RNA.

To analyze how band 1 is further processed, we labelled the 5' end of the RNA-I and incubated the samples with rDICER (see Figure 4A). Time course experiments and cloning results indicated that rDICER could process the 5'-labelled RNA-I at the 29-nt position from its 5' end (band 1) after 20 min incubation and subsequently cleave the 29-nt short RNA at the 6-nt position from its 5' end to 23-nt RNA (band 2) after 40 min incubation (see Figure 4A, 4B, and Table 1). This suggests dsRNAs with a 29 nt-5' strand and a 23 nt-3' strand are processed by rDICER from RNA-I at the first cleavage and released once from the enzyme. After this, rDICER binds the dsRNA again and, measuring from the 3' end of the 29-nt strand, generates 23-22 nt RNA duplexes via a second cleavage reaction (see Figure 5).

In this research, we found that hairpin RNA with trinucleotide-5' overhangs was cleaved into a 23-22 nt RNA duplex through two-step processing by rDICER. This could not be detected if we used only end-labelled RNA or label-incorporated RNA as a substrate for rDICER as reported previously [27,29]. In the first step, rDICER processes the hairpin RNA with 5' overhangs to dsRNA with 29 nt and 23 nt. Our results indicate the first processed dsRNA binds again in the inverse direction with the same or a different rDICER molecule and is again effective cleaved. The results are consistent with the previous report that human DICER protein binds either 3' ends of dsRNA strand on the PAZ domain and cleaves dsRNA at the ~23 nt position from the binding end [25]. In the sequential process described here, dsRNAs with 29 nt and 23 nt gradually increased and then stabilized at a steady level, followed by rapid increase of 23-22 nt duplexes (see Figure 3B and 3C). This indicates that dsRNAs with 29 nt and 23 nt are processed at a constant rate and 23-22 nt duplexes are belatedly processed at a similar rate. This suggests that the binding and processing of miRNA duplexes and releasing of the duplex from rDICER occur at the same rate. The bidirectional binding of processed dsRNA by DICER could result in directional presentation of dsRNA to Argonaute [16-18].

### The effect of 5' overhangs in substrate cleavage by recombinant DICER protein

We generated three different hairpin RNAs based on pre-hsa-mir-21 RNA: pre-mir-21 RNA, RNA-I and RNA-II (see Figure 2A, 3A and Additional file 1: Figure S1A, respectively). RNA-II is the same as pre-mir-21 RNA except for 5' addition. Using probe-1 to detect the cleavage product from the 5' strand for Northern blotting, both bands of mature miRNA in Figure 2B and band 1 (first cleavage product) in Figure 3B emerged after 20-min incubation and increased gradually. On the other hand, band 8 (probable first cleavage product) in Figure S1B was detected faintly after 30-min incubation but increased quite slowly (see Additional file 1: Figures S1B and S1D). Band 9 in Figure S1C was also detected in a similar manner as band 8 (see Additional file 1: Figure S1C). Our results indicated that longer 5' overhangs with stable stem structures could reduce the efficiency or rate of substrate cleavage.

### Processing of an endogenous hairpin RNA with 5' overhangs, pre-mmu-mir-1982 RNA, by recombinant DICER protein

**Figure 6:**
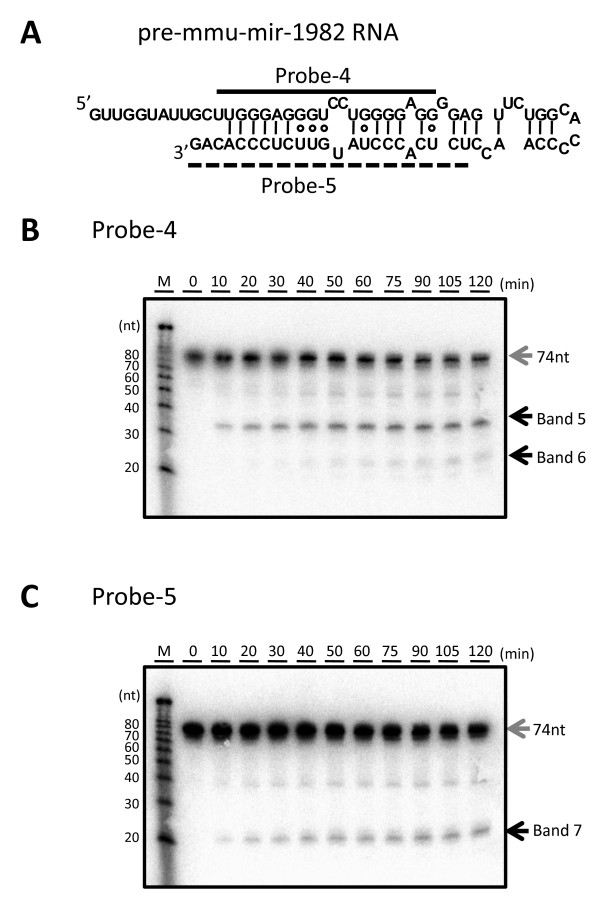
**Processing of pre-mmu-mir-1982 RNA by recombinant DICER protein**. (**A**) Pre-mmu-mir-1982 RNA and probes used in this study. The secondary structure was predicted using the CentroidFold program [49]. The solid line shows the position of probe-4 and the dashed line shows the position of probe-5. (**B-C**) Time-course analysis of the processing of pre-mmu-mir-1982 RNA by the rDICER protein. pre-mmu-mir-1982 RNAs were incubated with rDICER *in vitro *for the indicated time points (0, 10, 20, 30, 40, 50, 60, 75, 90, 105 and 120 min). The RNAs processed by rDICER were detected using probe-4 (**B**), probe-5 (**C**) by Northern blotting. The gray arrow shows the band of unprocessed RNA and the black arrow shows the bands of small RNA processed from the 5' strand and 3' strand of pre-mmu-mir-1982 RNA respectively. M: decade marker.

To demonstrate the processing ability of DICER protein with not only designed pre-miRNA with 5' overhangs but natural pre-miRNA with 5' overhangs, we performed the processing experiment using pre-mmu-mir-1982 RNA with rDICER. The cleavage products were detected by Northern blotting using two different probes, probe-4 and probe-5, corresponding to antisense sequences of bases 12-31 and bases 52-74 of pre-mmu-mir-1982 RNA, respectively (see Figure 6A). Using probe-4, band 5 (~35 nt) appeared after 10 min incubation and gradually increased. After 40 min incubation, band 6 (~23 nt) was detected and the abundance of band 6 increased in a time-dependent manner (see Figure 6B). Additionally, using probe-5, band 7 (~23 nt) was detected from the 10-min incubation sample (see Figure 6C). Pre-mmu-mir-1982 RNA can also be processed by *in vitro *rDICER activity.

**Figure 7:**
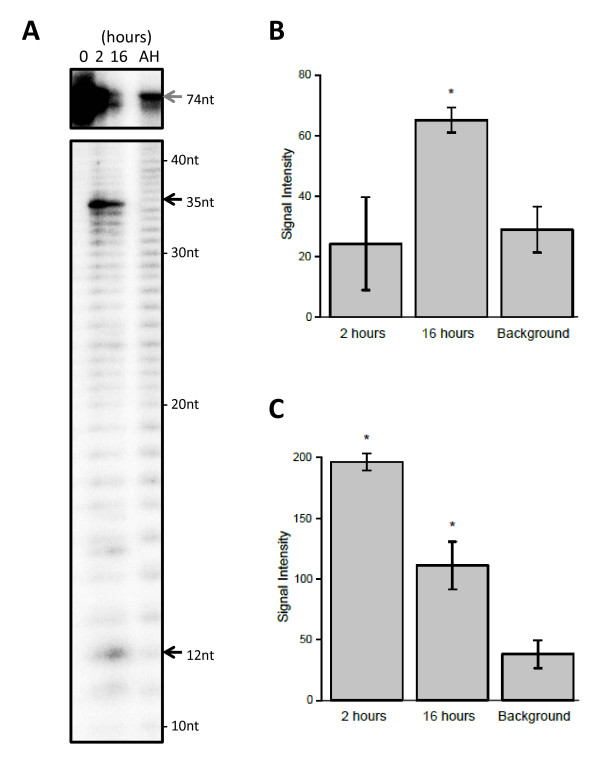
**Two-step processing of the 5'-end labelled pre-mmu-mir-1982 RNA by recombinant DICER protein**. (**A**) *In vitro *processing of the pre-mmu-mir-1982 RNA by the rDICER protein at a longer incubation time. 5' labelled pre-mmu-mir-1982 RNAs were incubated with rDICER for 0, 2 and 16 hours. The gray arrow shows the band of unprocessed RNA and the black arrow shows the bands of small RNA processed from pre-mmu-mir-1982 RNA. AH: the alkaline hydrolysis ladder of pre-mmu-mir-1982 RNA. The size of each band was determined by the AH ladder. (**B-C**) The signal intensities were quantified from the 12 nt (**B**) and 35 nt (**C**) bands in Figure 7A. These plots show average values bracketed by s.e.m. error bars; calculated from two independent experiments. Background refers to the signal intensity of the same sized band in the AH lane. The p-value was calculated using a simple t-test for each time point (2 hrs and 16 hrs) relative to the background. Significant differences (p < 0.05) in signal intensities are denoted with an asterisk. The significant calculated p-values are as follows: the 12-nt band at 16 hours, *p *= 0.017; the 35-nt band at 2 hours, *p *= 0.0073; and the 35-nt band at 16 hours, *p *= 0.024.

To analyze that band 5 could be further processed to band 6, we labelled the 5' end of the pre-mmu-mir-1982 RNA and incubated the samples with rDICER for 0, 2 and 16 hours (see Figure 7A). The signal intensity of 35-nt band decreased in a time-dependent manner, while on the other hand, the 12-nt band increased (see Figures 7B and 7C). These results showed that band 5 was a 35-nt product of initial rDICER processing and 12 nt from the 5'end were eliminated by a second, rDICER-catalyzed cleavage reaction generating a 23-nt product. Our results indicated that mature miR-1982 and miR-1982* RNA could be generated *in vitro *from pre-mmu-mir-1982 RNA by the two-step DICER processing reaction described above.

In addition to miRNA maturation, mammalian DICER also processes other kinds of small RNAs including endo-siRNAs [33,37-39]. It has been reported that mammalian endo-siRNAs are processed from various precursors including long hairpin RNAs and naturally formed dsRNAs resulting from bidirectional transcripts or antisense transcripts from pseudogenes. Although the complete structure of these precursor dsRNAs remains unclear, it seems likely that they have diverse 5' and 3' structures. Our results indicate DICER tolerance for 5' substrate overhang, potentially increasing the range of small RNA substrates that DICER can process. Recently, it was reported that the AGO2 protein could bind not only siRNAs and miRNAs but longer RNAs and pre-miRNAs [22,40]. However, most endogenous AGO2 proteins bind miRNAs [41] and the RISC requires 3' overhangs for the dsRNA incorporation [3,42]. As this research shows, DICER could process pre-miRNAs, longer dsRNAs and hairpin RNAs with 5' overhangs into dsRNA with 3' overhangs, which might be subsequently loaded with canonical length to the RISC. Further experimentation is required to connect our findings with the AGO loading mechanism.

## Conclusions

We show human rDICER recognizes and processes a hairpin RNA bearing a trinucleotide-5' overhang, and the two-step cleavage by rDICER forms canonical miRNA duplexes from the hairpin RNAs. It indicates that human rDICER functions as a molecular ruler by anchoring the 3' end of both the hairpin RNA with 5' overhangs and the 5' strand in the intermediate duplex. Moreover, an endogenously-expressed pre-miRNA with 5' overhangs, pre-mmu-mir-1982, also can be utilized as a substrate of rDICER and processed into a canonical miRNA duplex by the two-step cleavage reaction. While pre-mmu-mir-1982 RNA is a naturally expressed pre-miRNA [33,35], this 5'-overhanged structure is not a suitable substrate for nuclear export by Exportin-5 [43] and, assuming the absence of possible alternative export pathways, may not be presented to cytoplasmic DICER in the cells. However, it is worth noting a recent report, that mammalian DICER might be located in the nucleus and associate with ribosomal DNA chromatin [44]. We have also observed human DICER localized in both cytoplasm and nucleus (unpublished data, Ando *et al.*). These findings raise the intriguing possibility that nuclear DICER could process hairpin RNA with 5'-overhangs, like pre-mmu-mir-1982 RNA.

The two-step cleavage of a hairpin RNA with 5' overhangs shows that rDICER can release dsRNAs after the first cleavage and binds them again in the inverse direction for a second cleavage. The DICER protein's ability to release and bind dsRNA again indicates DICER could be capable of binding and processing dsRNA multiple times during short RNA maturation. DICER has recently been linked to the processing of diverse non-coding RNA precursors with as-yet undetermined structures. The experiments performed above suggest DICER has considerable flexibility in processing precursors, contributing to an ability to generate various short RNA products for incorporation into functional RISCs.

## Methods

### Preparation of hairpin RNA substrates

Pre-hsa-mir-21 RNA (pre-mir-21), pre-miRNA mimic hairpin RNA (RNA-I) and pre-mmu-mir-1982 RNA used in this study were generated by *in vitro *transcription using the Ampliscribe T7 High Yield Transcription kits (Epicentre) according to manufacturer's instructions. We made double-stranded DNA templates with T7 RNA polymerase promoter sequence by overlap-PCR using the following oligonucleotide pair; pre-mir-21-sense 5'-taatacgactcactatagAGCTTATCAGACTGATGTTGACTG-3' and pre-mir-21-antisense 5'-ACAGCCCATCGACTGGTGTTGCCATGAGATTCAACAGTCAACATC-3', RNAI-sense 5'-taatacgactcactatagggTGTCGGGTAGCTTATCAGACTGATGTTGA-3' and RNAI-antisense 5'-TGTCAGACAGCCCATCGACTGGTGTTGCCATGAGATTCAACAGTCAACA-3', pre-mmu-mir-1982-sense 5'-taatacgactcactataGTTGGTATTGCTTGGGAGGGTCCTGGGGAGGGGAGTT-3' and pre-mmu-mir-1982-antisense 5'-CTGTGGGAGAACATAGGGTGAGAGGTTGGGGTGCCAGAACTCCCCTCCCCA-3'. The overlapped sequences are underlined and the lower-case characters show the sequence of the T7 RNA polymerase promoter. *In vitro *transcription reactions were performed at 37°C overnight. Transcripts were run on 10% denaturing polyacrylamide gels in 0.5x TBE (45 mM Tris-borate, 1 mM EDTA), gel-excised, eluted from the gel in 1 M NaCl at 4°C overnight, and precipitated with ethanol. The pellet was resuspended in an appropriate volume of water and stored into the freezer at -30°C. Before use, RNA substrates were heated to 70°C for 5 min and then slowly cooled to room temperature.

### Affinity purification of recombinant FLAG-DICER fusion proteins

We assembled a full-length cDNA of human DICER1 protein from HeLa total RNA. This cDNA sequence was identical to the coding sequence cited in the Swiss-Prot Protein Database (Swiss-Prot) [Swiss-Prot: Q9UPY3]. N-terminally FLAG-tagged human DICER1 protein was purified from 293T cells transfected with the plasmid pCA-FLAG-DICER1. This vector contained the full-length human DICER1 protein FLAG-tagged at the amino terminus in a pCA-FLAG-DEST vector [45]. We purified the recombinant FLAG-DICER1 fusion protein (rDICER) using ANTI-FLAG M2-Agarose Affinity Gel (Sigma) and eluted by 0.1 M Glycine-HCl (pH3.5). Then, the eluate was neutralized by Tris-HCl (pH8.0). The average yield was 50-100 μg of the active form of rDICER protein from 1 × 10^8 ^culture cell. Purified rDICER protein was detected by Coomassie Brilliant Blue (CBB) staining and Western blotting using anti-DICER (H212, Santa Cruz) antibody to check for successful homogenous purification (see Figures 1A and 1B). The contamination of known DICER-binding proteins in rDICER samples was checked by Western blotting using anti-FLAG (M2, Sigma), anti-AGO2 (07-590, Upstate) and anti-TRBP (ab42018, Abcam) antibody, respectively (see Figure 1C).

### Processing of RNA substrates using recombinant DICER enzyme

The affinity-purified rDICER protein (2 pmol) was incubated with 45 pmol of RNA substrates (pre-mir-21 RNA, RNA-I or pre-mmu-mir-1982 RNA) in 1x reaction buffer (300 mM NaCl, 50 mM Tris-HCl, 20 mM HEPES, 5 mM MgCl_2_, pH 9.0) and 40 units of RNaseOUT (Invitrogen). These mixtures were incubated at 37°C for the indicated times. The reactions were purified by phenol-chloroform extraction followed by sodium acetate-ethanol precipitation at -20°C. The RNA pellet was resuspended in water at a final concentration of approximately 1 pmol/μl.

### Northern blotting

rDICER-processed RNAs (1 pmol) were separated on 7 M urea-denaturing 20% polyacrylamide gels, then blotted onto Hybond-N+ membranes (GE Healthcare) using a Trans-Blot SD Semi-Dry Transfer Cell (Bio-Rad). Hybridization was performed in Church buffer (0.5 M NaHPO_4_, pH 7.2, 1 mM EDTA and 7% SDS) containing 10^6 ^c.p.m./ml of each ^32^P-labelled probe for 14 h. The membranes were washed in 2x SSC, and the signals were detected by autoradiography. All experiments were repeated and replicated consistently.

The probe sequences in this study were as follows: probe-1 (5'-TCAACATCAGTCTGATAAGCTA-3'), probe-2 (5'-ACAGCCCATCGACTGGTGTTG-3'), probe-3 (5'-CCATGAGATTCAACAG-3'), probe-4 (5'-CCTCCCCAGGACCCTCCCAA-3') and probe-5 (5'-CTGTGGGAGAACATAGGGTGAGA-3'). The probes were 5'-end labelled using T4 polynucleotide kinase (TaKaRa Bio) with [γ-^32^P] ATP (6000Ci/mmol) at 37°C for 4 h.

### Cloning of cleavage products

rDICER-processed RNAs (1 pmol) were separated on 7 M urea-denaturing 15% polyacrylamide gels, then the gel was stained by SYBR Gold (Invitrogen). The band around 23 nt was excised from the gel and purified as described above. The purified RNA was cloned by the Small RNA cloning kit (TaKaRa Bio) and sequenced by capillary sequencing.

### 5'-end labelling of the transcript

For the 5'-end labelling, RNA (45 pmol) was dephosphorylated with CIP at 37°C for 60 min. The reaction was inactivated by phenol-chloroform extraction and precipitated by sodium acetate-ethanol at -20°C. The pellet was resuspended in an appropriate volume of water. The dephosphorylated transcript was 5' end-labelled using T4 polynucleotide kinase (TaKaRa Bio) with [γ-^32^P] ATP (3000Ci/mmol) at 37°C for 4 h. The 5'-end labeled transcript was PAGE-purified as described above and the RNA pellet was resuspended in water at a final concentration of approximately 0.5 pmol/μl. One microliter of this was used for the processing reaction by rDICER. These processed samples were run on 7.5 M urea-denaturing 20% polyacrylamide gels in 1x TBE buffer with RNA molecular marker or the products of alkaline hydrolysis of the same RNA molecule. The alkaline hydrolysis ladder was generated by incubating the labelled RNA in alkaline hydrolysis buffer (Ambion) at 100°C for 10 min. The signals were detected by autoradiography and quantified using ImageJ software (National Institutes of Health; http://rsb.info.nih.gov/ij/). The signal intensities were calculated as the mean of pixel value of selected area.

## Authors' contributions

YA conceived the study, designed and performed experiments and drafted the manuscript. YM and AM participated in the experimental design and performed experiments. RK and JC participated in the experimental design and purified recombinant FLAG-DICER fusion proteins. AMB, HS and KM participated in the design of the study and revised the manuscript. YH designed the research project, provided funding, supervised the study and critically reviewed the manuscript. All authors read and approved the final manuscript.

## Supplementary Material

Additional file 1 Supplementary information Click here for file
