# Supplementary material for: Two-step cleavage of hairpin RNA with 5' overhangs by human DICER
Source: BMC Mol Biol. 2011 Feb 9;12:6. doi: 10.1186/1471-2199-12-6 (PMC3048551; doi:10.1186/1471-2199-12-6)
Supplement: Additional file 1 — Supplementary information [file 1471-2199-12-6-S1.PDF]

# Table of contents

I. Supplementary methods

II. Supplementary figure legend

III. Supplementary references

IV. Supplementary figure

# I. Supplementary methods

## Preparation of hairpin RNA substrates

Pre-miRNA mimic hairpin RNA (RNA-II) used in this study was generated as described in *Methods*. Double-stranded DNA templates with T7 RNA polymerase promoter sequence was prepared by overlap-PCR using the following oligonucleotide pair; RNAI-sense 5'-taatacgactcactatagggTGTCGGGTAGCTTATCAGACTGATGTTGA-3' and pre-mir-21-antisense 5'-ACAGCCCATCGACTGGTGTTGCCATGAGATTCAACAGTCAACATC-3'. The overlapped sequences are underlined and the lower characters show the sequence of the T7 RNA polymerase promoter. *In vitro* transcription reactions were performed at 37$^{o}$C overnight. Transcripts were purified as described in *Methods*.

## Processing of RNA substrates using recombinant DICER enzyme

The affinity-purified rDICER protein (2 pmol) was incubated with 45 pmol of RNA substrate (RNA-II) in 1x reaction buffer (300 mM NaCl, 50 mM Tris-HCl, 20 mM HEPES, 5 mM $MgCl_2$, pH 9.0) and 40 units of RNaseOUT (Invitrogen). These mixtures were incubated and purified as described in *Methods*.

### Northern blotting

Northern blotting experiment was performed as described in *Methods*. The probe sequences in this study were as follows: probe-1 (5'-TCAACATCAGTCTGATAAGCTA-3') and probe-2 (5'-ACAGCCCATCGACTGGTGTTG-3'). The probes were 5'-end labelled as described in *Methods*.

### 5'-end labelling of the transcript

Labelled RNA-II (45 pmol) was prepared as described in *Methods*. Processed samples were run on 7.5 M urea-denaturing 20% polyacrylamide gels in 1x TBE buffer with RNA molecular marker and the products of alkaline hydrolysis of the same RNA molecule. The alkaline hydrolysis ladder was generated by incubating the labelled RNA in alkaline hydrolysis buffer (Ambion) at 100$^{o}$C for 10 min. The signals were detected by autoradiography.

# II. Supplementary figure legend

### Figure S1 - Processing of the hairpin RNA with 5' overhangs (RNA-II) by recombinant DICER protein.

(**A**) Hairpin RNA with 5' overhangs (RNA-II) and probes used in this study. RNA-II was a hairpin RNA with 9-nt 5' overhangs based on the "pre-mir-21 RNA" sequence.

The secondary structure was predicted using the CentroidFold program [1]. The solid line shows the position of probe-1 and the dashed line shows the position of probe-2; these probes were designed to detect the RNA sequences containing each position.

(**B-C**) Time-course analysis of the processing of RNA-II by the rDICER protein. RNA-II RNAs were incubated with rDICER *in vitro* for the indicated time points (0, 10, 20, 30, 40, 50, 60, 75, 90, 105 and 120 min). The RNAs processed by rDICER were detected using probe-1 (**B**), probe-2 (**C**) by Northern blotting. The gray arrow shows the band of unprocessed RNA and the black arrow shows the bands of small RNA processed from the 5' strand and 3' strand of RNA-II respectively. M: decade marker.

(**D**) *In vitro* processing of RNA-II by the rDICER protein at a longer incubation time. 5' labelled RNA-II RNAs were incubated with rDICER for 0, 2 and 16 hours. The gray arrow shows the band of unprocessed RNA and the black arrow shows the bands of small RNA processed from RNA-II. AH: the alkaline hydrolysis ladder of RNA-II. The size of each band was determined by the AH ladder. The cleavage site of first cleavage product on the 5' strand of RNA-II (35 nt) was different from the site of RNA-I (29 nt).

## III. Supplementary references

1. Sato K, Hamada M, Asai K, Mituyama T: **CENTROIDFOLD: a web server for RNA secondary structure prediction**. *Nucleic Acids Res* 2009, **37**(Web Server issue):W277-280.

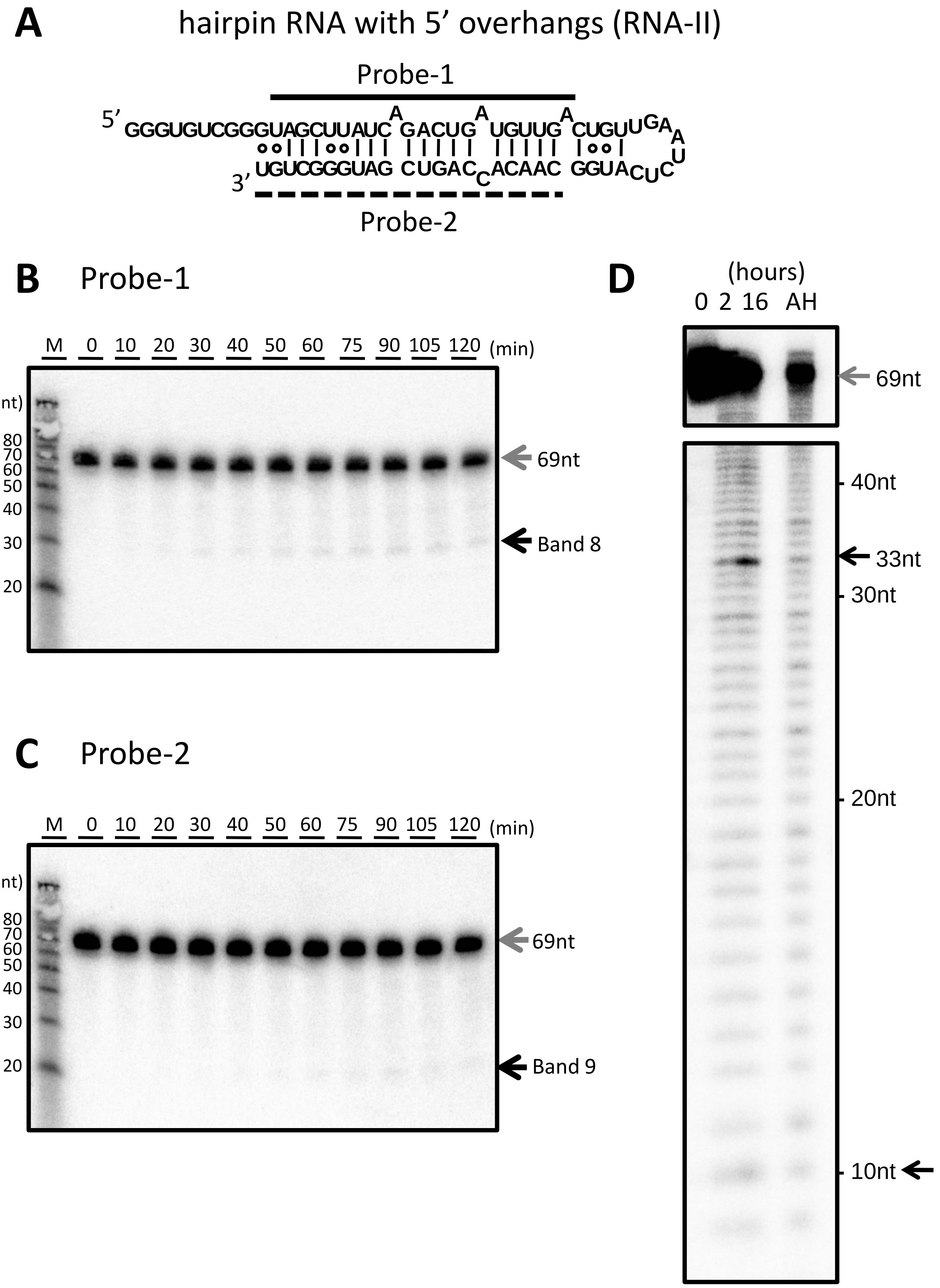
A
hairpin RNA with 5' overhangs (RNA-II)
Probe-1
Probe-2
B
Probe-1
M 0 10 20 30 40 50 60 75 90 105 120 (min)
(nt)
80
70
60
50
40
30
20
69nt
Band 8
C
Probe-2
M 0 10 20 30 40 50 60 75 90 105 120 (min)
(nt)
80
70
60
50
40
30
20
69nt
Band 9
D
(hours)
0 2 16 AH
69nt
40nt
33nt
30nt
20nt
10nt
